# Human Histology, in Its Relations to Descriptive Anatomy, Physiology, and Pathology

**Published:** 1857-12

**Authors:** 


ART. IV.—*Human Histology, in its Relations to Descriptive Anatomy, Physiology, and Pathology. With four hundred illustrations on wood.* By E. R. PEASLEE, M. D., Professor of Physiology aud Pathology in the New York Medical College; of Anatomy in Dartmouth College; and of Surgery in the Medical School of Maine, etc., etc. Philadelphia: Blanchard & Lea. 1857.

The work before us is the first elaborate treatise on histology that has been presented to the profession by an American author. Since the appearance of Bichat's great work about a quarter of a century ago, the novel and inviting field of histological research has been eagerly occupied by men of talent in all countries. America, however, appears to have cultivated this branch less than any other nation; it is now but a short time since microscopic anatomy and the application of the microscope to practical medicine and surgery, have been regularly taught in our medical schools; and though numerous works have appeared in England, and in the French and German languages, 1857 sees the first kindred publication in this country. The work of Kölliker, translated by George Busk and Thomas Graham, and edited by Dr. J. Da Costa, of Philadelphia, which is well known, doubtless, to all our readers, has become the standard work on microscopic anatomy; this however, is merely a work on microscopic anatomy and nothing more. The work of Robin and Verdeil, on anatomical and physiological chemistry (chimie anatomique) which has been lately issued, and is not yet translated, is, perhaps, the most elaborate and complete treatise on the subject, in any language. This treats of the anatomical and physiological chemistry of the tissues or the normal and pathological immediate principles of the human body and mammalia. Dr. Peaslee has availed himself largely of these works, and of Lehmann's Physiological Chemistry in the preparation of this volume.

In his own words his aim is:

"I. *To give a connected view of the simple chemical elements, of the immediate principles, the simple structural elements, and the proper tissues entering into the composition of the fluids and solids of the human body.*

"II. *To associate with the structural elements and the tissues, their function while in health and the changes they undergo in disease.*"

"In the prosecution of his object, the author has drawn more especially upon the work of Robin and Verdeil in the first part; that of Lehmann in in the division including the fluids; and on Kölliker's excellent work in the part on the tissues proper. He has not hesitated, indeed, sometimes to use the language of the latter, when sufficiently concise. He has not, however, embarrassed the text by references to the various authorities whence materials have been derived; except in instances where views varying from those of previous writers are advanced, as being, in the author's judgment, the most satisfactory at the present time."

The work is divided into two parts. Part I, treating of Stæchiology, or the description of elements; and Part II, of Histology proper. The first divion of Part I, merely enumerates the elements which compose the human body; the second division, however, treats of the immediate principles. The definition of immediate principles is taken from Robin and Verdeil; they (immediate principles) are "the last bodies constituting the organism to which the tissues can be reduced by mere *anatomical analysis;* and which admit of no further subdivision without *chemical decomposition.*"

The entire consideration of the anatomical elements or immediate principles of the human organism, is embraced in about seventy pages. Though the author says in the preface that he has drawn upon the works of Robin and Verdeil and of Lehmann, the reader will hardly be prepared for the extent to which these authorities (especially Robin and Verdeil) have been made to contribute. The classification of Dr. Peaslee is identical with that in the *chimie anatomique*, with the exception of the omission of a few unimportant principles; but the consideration of these elementary principles is the merest skeleton of a description, and it seems to us cannot be of much greater use to the student than a mere enumeration. The following is the classification of Dr. Peaslee:*

*Tabular Classification of the Immediate Principles.*

GROUP I. Principles Crystalizable or Volatile, Independently of Decomposition.

FIRST CLASS. Principles of Mineral Origin. (24.)

*First Division.* Gaseous and not Saline. (5.)

| | |
|---|---|
| Oxygen, | Carbonic acid, |
| Hydrogen, | Water. |
| Nitrogen, | |

* Dr. Peaslee reckons 84 immediate principles as determined; and in a foot note states that Robin and Verdiel reckon 92; this is a mistake, R. and V. reckon 96.—See Chimie Anatomique, vol· i, page 130.

*Second Division.* Salts. (19.)

| | |
|---|---|
| Chloride of Sodium, | Sulphate of Soda, |
| " " Potassium, | " " Lime, |
| Fluoride of Calcium, | Basic Phosphate of Lime, (Bones), |
| Hydrochlorate of Ammonia, | Acid " " " |
| Carbonate of Lime, | Phosphate of Magnesia, |
| " " Magnesia, | Neutral Phosphate of Soda, |
| " " Potassa, | Acid " " " |
| " " Soda, | Phosphate of Potassa, |
| Bicarbonate " | Ammonio-Magnesian Phosphate. |
| Sulphate of Potassa, | |

SECOND CLASS. Principles of Organic Origin formed within the Body by Dis-Assimilation. (42).

*First Division.* Acid or Saline Principles, (23).

| | |
|---|---|
| Lactic Acid, | Hippuric Acid, |
| Lactate of Soda, | Hippurate of Lime, |
| " Potassa, | " " Soda, |
| " Lime, | " " Potassa, |
| Oxalate of Lime, | Inosate of Potassa, |
| Uric Acid, | Pneumic Acid, |
| Neutral Urate of Soda, | Pneumate of Soda, |
| Acid " " | Taurocholate " |
| Urate of Potassa, | Hyocholinate " |
| " Magnesia, | Glychocholate " |
| " Lime, | Lithofellic Acid. |
| " Ammonia, | |

*Second Division.* Neutral Nitrogenized Compounds. (5.)
(Nitrogenized *Alkaloids.*)

| | |
|---|---|
| Creatine, | Urea (and chloro-sodate of urea—Urea with marine salt,) |
| Creatinine, | Cystine. |

*Third Division.* Neutral Non-nitrogenized Compounds. Sugars, (2.)

| | |
|---|---|
| Sugar from the Liver, | Sugar of Milk. |

*Fourth Division.* Fatty and Saponaceous Compounds. (13.)

| | |
|---|---|
| Cholesterine, | Caproate of Potassa, Soda, etc., |
| Oleic Acid, | Oleine, |
| Margaric Acid, | Margarine, |
| Stearic " | Stearine, |
| Oleate of Soda, | Elaterine, |
| Margarate " | Stearine. |
| Stearate " | |

GROUP II. Principles Non-crystalizable or Non-volatile, Independently of Decomposition.

THIRD CLASS. Organic Substances or Coagulable Principles. (18.)

*First Division.* Those Naturally Liquid. (7.)

| | |
|---|---|
| Fibrine, | Pancreatine, |
| Albumen, | Mucosine, |
| Albuminose, | Ptyaline, |
| Caseine, | |

*Second Division.* The Solid and Demi-solid. (7.)

| | |
|---|---|
| Globuline, | Cartilagine, |
| Crystaline, | Osteine, |
| Musculine, | Keratine. |
| Elasticine, | |

*Third Division.* Pigmentary Substances. (4.)

| | |
|---|---|
| Hematine or Hematosine, | Melanine, |
| Biliverdine, | Unosacine. |

The author adds to this an enumeration of "Certain immediate principles of probable or certain existence, though not well determined, and substances known to exist, but doubtful as immediate principles."*

The author then proceeds to a consideration of the individual immediate principles according to the classification. In treating of carbonic acid he quotes the statement of Robin and Verdeil that there is actually more of this gas in

* Quoted from Robin and Verdeil.

solution, in the arterial than in the venous blood; this fact is not generally known to the profession of this country, and is disputed by most of the English and American physiologists; it is in direct opposition to the generally received and taught doctrines, and therefore would seem to merit at the author's hands something more than a mere statement. We have such an array of distinguished authority against it, that in a work like this, we would expect to be told the grounds on which this assertion is based; the author leaves the subject, however, without the smallest sustaining argument. This barrenness of detail characterizes the whole of Part I.

Passing over the consideration of the remaining articles of the first class, and the articles of the second class, (which are treated of very briefly,) we come to articles of the third class, *i. e.* Organic Substances, or Coagulable Principles. This is by far the most important class of elements, and receives a correspondingly lengthy consideration, though by no means sufficiently extended. In treating of fibrin, the author's views do not seem to be modeled upon the opinions of Robin and Verdeil, as in other instances, or indeed upon the doctrine taught by the most advanced physiologists of the day. In speaking of coagulation he says, "Coagulation is fibrillation, and is to be regarded as a vital act, since it is organization, though, perhaps, of the lowest kind. But fibrillation is the last and highest vital act of the fibrin." Robin and Verdeil take the ground, and in our opinion sustain it, that coagulation is not a vital act, and that mere coagulated fibrin is never organized. Vital processes only go on within the organism, and the coagulation of fibrin *never* takes place under normal conditions, but either when it is removed from the vessels or when the circulation is arrested; mere motion is not sufficient to prevent the coagulation of fibrin, for we find that it readily deposits itself about a bundle of twigs agitated in freshly drawn blood. The fibrin in the vessels, in healthy circulation, is in its normal condition, and remains fluid; but when we remove it from its normal situation, this removal has the same effect as heat upon albumen, it coagulates it. The mere fact of fibrillation during coagulation, is no argument in favor of its being a vital act, and the commencement of organization. Is there any reason why fibrin should not have a tendency to coagulate in a particular form, as well as that the triple phosphate should have a tendency to resolve itself into prisms? And yet Dr. Peaslee certainly would not assert that the crystalization of the triple phosphate was the commencement of organization, and we can easily distinguish fibrils of fibrin from proper areolar tissue, or any other tissue, as crystals of the triple phosphate from a muscular fibre.

We have already been betrayed into too lengthy a consideration of Part

I, and now pass to Part II, which presents a more inviting appearance. We hope ere long to see a translation of the work which has been made use of so largely by Dr. Peaslee, but in the mean time would recommend those who are not satisfied with this meagre compendium, to consult it in the French; they would then be able to see how little justice has been done to the French authors, and how clearly every point, which is here merely stated, is elaborated and sustained by sound observation and deduction.

Part II consists of three divisions:

First. "A description of the simple histological elements, their distribution, development, &c."

Second. "Of the fluids in the human body which contain histological elements."

Third. "Of the tissues properly so-called."

Parts second and third may be deemed as really embodying the substance of the book, and from the simple arrangement and division, as well as for other merits as a text book, will be of great value to the medical student. The work of Kölliker is more elaborate, and better adapted to the wants of one further advanced in histological investigation; it treats only of healthy microscopic anatomy, while the work of Dr. Peaslee, at the same time considers pathological histology.

The bulk of the first division of Part II is occupied by a consideration of cells, or cytology. This subject is discussed at length, and the views of cell development which are now generally received, are here put forward. Free cell development is first considered, that is, the development of cells in a clear plasma. The author seems to us to be a little obscure in his arguments in this connection, but at all events, he arrives at the conclusion, in regard to the formation of cells: "That no sufficient reason appears why a primary utricle may not be, in all cases, as easily formed in clear plasma, as a nucleus can; and if this becomes a cell in one instance, and then forms a nucleus *within* itself, it is hardly probable that a nucleus on the other hand, is first formed in other instances, and then forms a cell *around* itself. It is far more probable that the first cells are formed in a clear plasma without the aid of a nucleus, they being merely fully developed utricles; true *free cell* formation always proceeding in this way. The second generation of cells may be formed from the nuclei in the first, or not; but if so it is endogenous formation and not the form of development here under consideration."

It seems to us that there *is* a sufficient reason why it is more probable that cells are formed subsequent to the nuclei if not from them, than that nuclei are formed after the development of cells. A cell, according to Dr. Peaslee's own definition in the preceding page,) consists of a cell wall, a contained fluid, granules floating in the fluid, a nucleus and nucleolus. Now if the cells were first developed and then the nucleus, we should expect often to see cells without nuclei, or cells which had not had sufficient time to form their nuclei; but in support of the other side of the question we have the fact that we often see great quantities of free nuclei, both in health and in morbid structures, while we much more rarely see a cell in which there is no nucleus.

The subject of cell development is full of obscurity, and if taking the ground of some of the French positive philosophers, that we were to believe only what can be demonstrated, we should be compelled to acknowledge ourselves unable to say whether a cell elongated and became a fibre, or whether a fibre contracted and became a cell. Some of the most distinguished French microscopists have reduced the class of cells in the organism to a very small space, regarding those only as cells in which a membrane can be demonstrated, and this becomes a difficult matter in cells the size cf a blood disc or a tubercular corpuscle.*

The author finishes the division on cells by a consideration of the cancer cell, which has been the cause of so much controversy within the last few years. He takes the ground that "in all cases of *well developed* cancerous formation, the microscope, in the hands of one *skilled in its use*, will alone demonstrate the true character of the growth, unaided by any knowledge of the appearance to the naked eye, of its tactile properties, or of the history of the case." We are surprised at any modern writer taking such decided ground as this; giving to the microscope what its most earnest defenders and the most expert proficients in its use, have ceased to claim for it; we have ourselves repeatedly examined specimens of tumors which were undoubtedly of a cancerous character, and failed, after repeated and patient endeavors, to find the cancer cell. We cannot claim, however, to be sufficiently skilled in its use to present this alone as an argument against Dr. Paislee's assertion; but we know that this is the experience of some of the most skillful and reliable microscopists in the country, one of whom, espe-

* The views of Robin on this subject may be found in the Dictionnaire de Médicine, under the head of cellule. He regards all the vegetable tissues as cellular and formed by cells, and also that the ovum is developed in the same way ; but in the fully developed human body, he is of the opinion that a great many things have been called cells which have not the first element of a cell, i. e. the cell wall; and that they are merely semi-solid masses of matter.

cially we can recollect searching repeatedly for the cancer cell, in a growth about which there could be no manner of doubt as to its cancerous character, and which eventually killed the patient, though the cancer cell could never be discovered.

The second division of Part II treats of the animal fluids. It gives a clear and distinct account of the blood, lymph, chyle, serous secretions, transudations, exudations, pus, mucus, gastric and intestinal fluids, and all the fluids of the human organism. The anatomy of the fluids, however, is by far the most elaborately considered, though we have at the same time a brief sketch of their physiological relations and some of their pathological changes.

The third and last division, comprising more than half the entire volume, is devoted to a consideration of the tissues proper. Beginning with epithelium, the author describes the various varieties, their mode of development, physiological uses, dimensions and distribution, with some of their pathological changes; especially as seen in the disease known as epithelial cancer; the remainder of the chapter is occupied by a similar consideration of the hair and nails.

We have not the space to follow the author closely through the descriptions of fibrous tissue, areolar tissue, adipose tissue, etc., but can merely state that the same plan is adopted in the consideration of these, as of the preceding tissues, i. e. we have the anatomy of the tissue, a sketch of its development, physiological uses and pathological changes. As both the physiology and development, and the pathological changes are in themselves sufficient to occupy a large volume in their elaborate consideration; in such a work as we have before us, we could not expect, nor do we have, anything more than a mere outline of these portions of the subject.

In speaking of fatty degeneration, we are glad to see the author giving a clear and unmistakable definition of the term. The term itself implies an actual degeneration of organs or parts of the body into fat. This, however, is not the fact, "An organ in a state of fatty degeneration is one, some or all of whose structural elements have been *replaced by fat in the form of globules or granules*, and not inclosed in fat cells." This is the true process which goes on in the so-called fatty degeneration of various organs of the body, as the liver or heart; the normal anatomical element of the organ is absorbed, and instead of being replaced by the same substance as in health, is replaced by fat. The term "fatty replacement or substitution" then, would be infinitely preferable to "fatty degeneration," but the difficulty of substituting a new term, though it be exactly expressive of the process which we wish to describe, for one which has been long in general use, is almost insurmountable.

We would gladly follow out the remainder of this work, especially as we have dwelt on the least attractive and meritorious portion, namely, Part I; we were betrayed into such an extended notice of that portion, for the reason that it contained many of the views of Robin, (whom we regard as one of the most brilliant lights of the profession in any country,) and other writers which have never received much attention in this country, and many of which are not even known. We are glad to see them thus brought before the profession, but at the same time regret that they are not entered into more fully by the author of the present volume. The discovery of pneumic acid, for example, by Verdeil, with its bearings on the physiology of respiration and the interchange of gases in the lungs, presents a field which should be somewhat occupied even in a volume of this character. The peculiar views of Robin on cells are not even mentioned, and being but little known to Americans, this would have been an admirable opportunity of bringing them forward and discussing their merits. Still, all these questions could not have been more fully elaborated without materially augmenting the size of the volume, which would have made it appear more formidable to the medical student, for whom it is especially intended. We think it would have been better to have omitted Part I entirely, and elaborated more fully the pathological sections of Part II. It is much more easy to elaborate than to abridge, and we can easily conceive how difficult a task it has been to condense into seventy pages, matter which is sufficient to occupy three large volumes. If the ground which Dr. Peaslee attempted to occupy were less broad, we conceive that he would have made a much more useful and acceptable book.

The purely histological portion, however, seems in every way adapted to the wants of the student, and is in a much better form than any we have yet seen. The views are sound and ably sustained, and the description sufficiently minute for the purpose. Among the wood cuts we recognize the faces of many a familiar friend which has contributed to illustrate numerous other works on this subject.

In conclusion, we would state as our general opinion of the work, that as a text-book, and a companion to lectures, it cannot fail to be exceedingly useful; and while the author certainly cannot claim any degree of originality, or even that he has represented the existing state of knowledge on the subjects of which he treats; yet it bears the impress of careful and judicious preparation, and in most instances, inculcates sound doctrine. F.

